# A classification of response scale characteristics that affect data quality: a literature review

**DOI:** 10.1007/s11135-017-0533-4

**Published:** 2017-07-24

**Authors:** Anna DeCastellarnau

**Affiliations:** 1 RECSM-Universitat Pompeu Fabra, Ramon Trias Fargas, 25-27, Mercè Rodoreda Building, 08003 Barcelona, Spain; 2 Tilburg University, Tilburg, The Netherlands

**Keywords:** Data quality, Measurement error, Literature review, Response scale characteristics, Classification

## Abstract

Quite a lot of research is available on the relationships between survey response scales’ characteristics and the quality of responses. However, it is often difficult to extract practical rules for questionnaire design from the wide and often mixed amount of empirical evidence. The aim of this study is to provide first a classification of the characteristics of response scales, mentioned in the literature, that should be considered when developing a scale, and second a summary of the main conclusions extracted from the literature regarding the impact these characteristics have on data quality. Thus, this paper provides an updated and detailed classification of the design decisions that matter in questionnaire development, and a summary of what is said in the literature about their impact on data quality. It distinguishes between characteristics that have been demonstrated to have an impact, characteristics for which the impact has not been found, and characteristics for which research is still needed to make a conclusion.

## Introduction

A challenge for questionnaire designers is to create survey measurement instruments (from now on called: survey questions) that capture the true responses from the population. To do so, they need to create survey questions that not only capture the theoretical concept under evaluation, but that also minimize the impact of their design characteristics on the quality of the responses.

Deciding about the right characteristics of a survey question is not a straightforward task. For instance, ‘What is the optimal number of response options to use?’ or ‘Shall I label all options in the scale? are recurrent questions without a clear answer in the field of questionnaire design and survey methodology. However, making the right decisions is crucial if one wants to minimize the impact of those on survey’s data quality (Alwin 2007; Dolnicar 2013; Krosnick 1999; Krosnick and Presser 2010; De Leeuw et al. 2008; Saris and Gallhofer 2014; Schuman and Presser 1981).

Within the Total Survey Error framework (Groves et al. 2009), the way a survey question is designed has a direct influence on the responses given to such question, and impacts the overall surveys’ data quality. The observational gap between the ideal measurement and the response obtained, is defined as measurement error. Studies assessing the influence of questions’ characteristics on measurements’ error show that these characteristics explain between 36 and 85% of its variance (Andrews 1984; Rodgers et al. 1992; Saris and Gallhofer 2007; Scherpenzeel and Saris 1997). Saris and Revilla (2016, p. 4) state that if measurement errors are ignored: “one runs the risk of very wrong conclusions with respect to relationships between variables and differences in relationships across countries”.

Among the wide range of components that influence the design of a survey question, the choice of the response scale is often the most important decision to assure good measurement properties. For instance, Andrews (1984) showed that the number of categories had the biggest effect on measurements’ quality, followed by the provision or not of an explicit “don’t know” option. Moreover, the design of the scale is often the most complex in terms of the amount of decisions that influence the way respondents interpret the options provided.

Literature on how to design scales is wide. Most research is directed to the study of a specific set of design characteristics, like the optimal number of points (Preston and Colman 2000; Revilla et al. 2014) or the kind of labels to use (Eutsler and Lang 2015; Moors et al. 2014; Weijters et al. 2010). Some literature reviews have been conducted to summarize all these findings (e.g. Dolnicar 2013; Krosnick and Fabrigar 1997; Krosnick and Presser 2010). However, these summaries focus on the most commonly used characteristics and do not provide an accurate guide of all design decisions that developing a scale can require. Moreover, one can get quite lost because of the different classification strategies and the different ways researchers use to refer to the same aspects.

In this paper, I aim to provide an updated and detailed classification of characteristics to be used in the development of scales in combination to their influence on data quality. Specifically, I focus on closed and ordinal response scales for forced-choice scales because, in contrast to multiple-choice, open and nominal scales, many more subjective design decisions can take place.

To make such a classification, I conducted a revision of the literature with two main objectives: (1) classify the characteristics of response scales, and (2) assess whether evidence has been found, in the literature, regarding the impact of those characteristics on data quality.

The reminder of this paper is organized in the following way: Sect. 2 presents the methodological procedure followed to review the literature and make the classification. Section 3 presents the findings from the literature review following the classification. And, finally, Sect. 4 concludes with the main findings of this research.

## Methodological procedure

I conducted a revision of the literature looking for evidence about the relevance of the characteristics of closed and ordinal response scales.

As a starting point, I took the list of characteristics developed by Saris and Gallhofer (2007) and further updated in Saris and Gallhofer (2014). They structured this list in characteristics which group different mutually-exclusive choices. For instance, the characteristic: *labels of categories*, groups three possible choices: *no labels*, *partially*-*labelled* or *fully*-*labelled*. In total, they considered more than 280 possible choices, among which 40 choices are related to the design of the scale and belong to 17 characteristics. Table 2 in Appendix provides the list of response scales’ characteristics and the choices considered by these authors. This list covers most characteristics used in the development of scales for face-to-face surveys, that used showcards as visual aid for the respondent. Its major drawback comes from specific characteristics related to the design possibilities offered by other modes of survey administration, such as the different formats of scales’ visual presentation which are available in web surveys. From this preliminary list, I conducted an in-depth search for publications that mention these 40 design choices in academic journals or book chapters.

While revising the literature I focused, on the one hand, on identifying other characteristics and design choices, and on the other hand, I searched for empirical evidence and/or theoretical arguments in the literature that assess if these design choices have an impact on data quality or not.

In relation to the empirical evidence, it is often difficult to extract general conclusions since studies differ on the type of questions under examination, on the sample characteristics, on the mode of administration, and especially on the type of quality indicators used. Moreover, there are clear dependencies between characteristics. However, in this paper my goal is to identify if there is any kind of empirical evidence in the literature, thus, I will not differentiate the study characteristics or on the sign of the effect found, or on the kind of indicators. In fact, a wide range of measurement quality indicators, or its complement measurement error, are considered in the literature. Hereafter I considered different types of response style bias, like extreme and middle responding and acquiescence, item non-response, and satisficing bias as indicators of measurement error. Furthermore, I considered different measures of reliability and validity, as indicators of measurement quality.

**Fig. 1:**
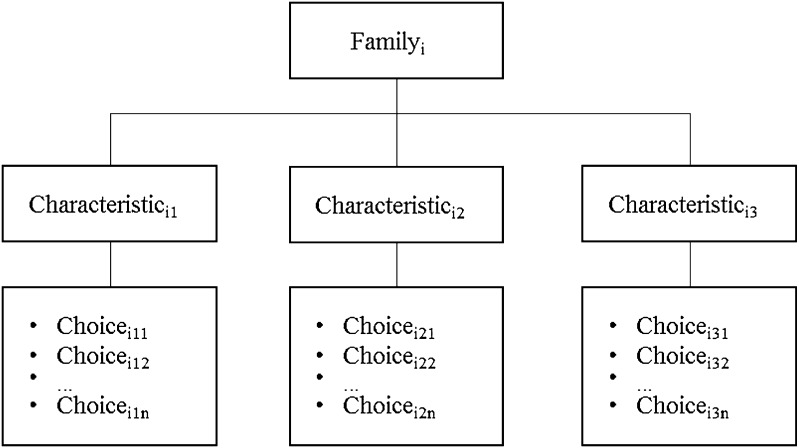
Three-level classification structure

The revised literature often uses different terms for the same types of design choices. To provide a clear summary of the literature review, an initial step is to harmonize the terminology. When necessary, I therefore renamed characteristics and add more possible design choices. I thereby also identified the gaps of non-studied variations that should also be considered. Subsequently, as illustrated in Fig. 1, I group within families, similar sets of related characteristics, and within a characteristic the different number of mutually-exclusive choices one could take.

Next, using this classification, I summarize the results of the literature review.

## The findings from the literature review

By the end of this process, I have reviewed 140 publications from which I have used 88, and from which I have identified 83 different design choices related to the design of response scales, i.e. 43 more than Saris and Gallhofer’s preliminary list. First, I classified those mutually-exclusive choices into 23 different characteristics. Finally, I have classified these into four main families of related characteristics. Table 1 presents this classification and provides information on the four possible scenarios regarding its impact on data quality: (1) whether a characteristic has been empirically demonstrated to have an impact on data quality (Yes); (2) whether it has been shown to not impact data quality (No); (3) whether it has not been studied (NS); or (4) whether its impact is not clear yet to make a conclusion (NC).

**Table 1:** Classification and impact on data quality of the complete list of characteristics and design choices

Characteristics	Design choices	Impact
*Characteristics of the scales’ conceptualization*		
Scales’ evaluative dimension	Agree–disagree	Yes
	Item-specific	
Scales’ polarity	Bipolar	NC
	Unipolar	
Concept-scale polarity agreement	Both bipolar	NC
	Both unipolar	
	Bipolar concept with Unipolar scale	
	Unipolar concept with Bipolar scale	
*Characteristics of the type of scales and length*		
Types of scales	Absolute open-ended quantifier	Yes
	Relative open-ended quantifier	
	Relative metric	
	Absolute metric	
	Dichotomous	
	Rating	
	Closed quantifiers	
	Branching	
Scales’ length	Minimum value	Yes
	Maximum value	
	Number of categories	
*Characteristics of the scales’ labels*		
Verbal labels	Fully-labelled	Yes
	End-points and more points labelled but not all	
	Endand midpoints points labelled	
	End-points only labelled	
	Not labelled	
Verbal labels’ information	Non-conceptual	NC
	Conceptual	
	Objective	
	Subjective	
	Full-informative	
Quantifier labels	Vague	NC
	Closed-range	
Fixed reference points	Number of fixed reference points	Yes
Order verbal labels	From negative-to-positive	No
	From positive-to-negative	
Nonverbal labels	Numbers	No
	Letters	
	Symbols	
	None	
Order numerical labels	Negative-to-positive	Yes
	Positive-to-negative	
	0-to-positive	
	0-to-negative	
	Positive-to-0	
	Negative-to-0	
	1 (or higher)-to-positive	
	Positive-to-1 (or higher)	
Correspondence between numerical and verbal labels	High	Yes
	Medium	
	Low	
Scales’ symmetry	Symmetric	NC
	Asymmetric	
Neutral alternative	Explicit	Yes
	Implicit	
	Not provided	
“Don’t know” option	Explicit	NC
	Implicit	
	Not provided	
*Characteristics of the scales’ visual presentation*		
Types of visual response requirement	Point-selection	No
	Slider	
	Text-box input	
	Drop-down menu	
	Drag-and-drop	
Slider marker position	Left/bottom	NC
	Right/top	
	Middle	
	Outside	
Scales’ illustrative format	Ladder	Yes
	Thermometer	
	Other	
	None	
Scales’ layout display	Horizontal	Yes
	Vertical	
	Nonlinear	
Overlap between verbal and numerical labels	Overlap present	NS
	Text clearly connected to categories	
Labels’ visual separation	Non-substantive options	Yes
	Neutral options	
	End-points	
	All points	
	None of the points	
Labels’ illustrative images	Feeling faces	No
	Other human symbols	
	Nonhuman symbols	
	None	

Following, a detailed description of each characteristic and design choices together with the findings related to their influence on data quality is provided using the classification presented in Table 1. The description below follows the detailed summary provided in the Table 3 in Appendix, which also provides all the theoretical and empirical references used as well as the indicators used to assess the impact on data quality for each study.

### The scales’ conceptualization

#### Scales’ evaluative dimension

The evaluative dimension of the scale comes from the theoretical underlying concept that is intended to be measured by the survey question. The basic distinction is between agree–disagree and item- (or construct-) specific scales.

*Agree*–*disagree scales* can be used to evaluate the level of agreement or disagreement towards a statement or a stimulus. For instance, asking “Do you agree or disagree that your health is good?” and providing the respondents with the options “agree” and “disagree”. Such type of scales has obtained a lot of attention by researchers. These scales are simple to design (Brown 2004; Schaeffer and Presser 2003) but they require a major cognitive effort from respondents (Kunz 2015). Empirical evidence has shown presence of acquiescence bias, i.e. the propensity to agree, in such scales (Billiet and McClendon 2000). *Item*-*specific scales* can be used to measure variables, for which the scale options directly refer to the theoretical concept under evaluation. For instance, when asking “How good or bad is your health?” an item-specific scale would provide the respondents with the options “good” and “bad”. Comparing item-specific with agree–disagree scales, studies have shown that item-specific scales provide higher measurement quality (Alwin 2007; Krosnick 1991; Revilla and Ochoa 2015; Saris et al. 2010; Saris and Gallhofer 2014). The choice of the scale’s evaluative dimension has therefore, an impact on data quality.

#### Scales’ polarity

Every concept has a theoretical range of polarity, which can be either bipolar or unipolar. While bipolar constructs range from positive to negative with a neutral midpoint; unipolar constructs range from zero to some maximum level with no neutral midpoint. Scales’ polarity refers to the conceptual extremes of the labels used in the scale. A *bipolar scale* uses the two theoretical poles of the bipolar concept being measured in the scales’ extremes, for instance, “satisfied” and “dissatisfied”. A *unipolar scale* uses only one pole of the concept being measured for one extreme and its zero point for the other, for instance, “important” and “not important at all”. This distinction is relevant, because in case a unipolar scale is used to measure a bipolar concept, the scale would be one-sided towards the positive or the negative pole. Moreover, it is important to consider since specific characteristics like the use of a midpoint or the use of a symmetric scale depend on whether the scale is provided as unipolar or bipolar. While bipolar scales ask about the neutrality, the direction and the intensity of an opinion, unipolar scales only ask about the extremity or intensity. Moreover, bipolar scales have the disadvantage that some respondents are reluctant to choose negative responses (Kunz 2015), and that reliability is somewhat higher in unipolar scales than bipolar scales (Alwin 2007). However, I have not found more studies assessing the impact of the scales’ polarity on data quality. Thus, more research is needed to confirm its relevance.

#### Concept-scale polarity agreement

The distinction between the concepts and the scales’ polarity is key, since the non-differentiation between bipolar and unipolar attributes has resulted in “misinterpretations of the empirical findings” (Rossiter 2011, p. 105). Even so, when designing survey questions, this characteristic has received quite little attention, compared to other aspects of the survey questions. It has been shown that this characteristic has an impact on the response styles (van Doorn et al. 1982) but no clear impact on measurement quality (Saris and Gallhofer 2007). Thus, more research is needed about its impact on data quality. Following the classification of Saris and Gallhofer (2007), the design of concept-scale polarity can be: *both bipolar*, *both unipolar*, or *bipolar concept with a unipolar scale.* In practise, even if, theoretically unipolar concepts should be designed using unipolar scales, we find also bipolar scales. For instance, a scale ranging from “Completely unimportant” to “Completely important” would be a *unipolar concept with a bipolar scale.* So far it was not studied whether it has or not an impact and whether the formulation of these scales affects their interpretation but we should account for this reality. I therefore propose to add this choice to the classification.

### The type of scale and its length

#### Types of scales

There are multiple types of continuous scales. I distinguish four main types: (1) *absolute open*-*ended quantifiers*, a type of numerical text input scale, used to ask respondents an open and numerical answer; (2) *relative open*-*ended quantifiers*, a similar type of numerical text input scale, which require a previous specification of the meaning of a standard value; (3) *relative metric scales*, a kind of scale that also requires the specification of a standard to give relative evaluations. However, in this case, respondents are asked to draw a line relative to the standard provided instead of giving a numerical answer; and (4) *absolute metric scales*, where respondents should select a point in a continuum. Typically, it is presented as a straight horizontal or vertical line with specified anchors on each end-point.

Rounding is the major problem of continuous numeric options. It has been shown that respondents create their own grouped response categories, often using exact multiples of 5 (Liu and Conrad 2016; Tourangeau et al. 2000), except for the relative metric scales which, in contrast, require lines’ length to be measured later (Saris and Gallhofer 2014). Relative scales are argued to be more burdensome to respondents which should not give an absolute evaluation but instead a relative answer given the standard value specified (Krosnick and Fabrigar 1997). Moreover, the specification of an appropriate standard is sometimes hard, since it is important using a standard that is “part of actual experience for all respondents” and “perceived as distinct from the 0 point” (Schaeffer and Bradburn 1989, p. 412). The impact on measurements’ error of using these types of scales has been studied by comparing absolute open-ended quantifiers with absolute metric scales with mixed results: Liu and Conrad (2016) find non-significant differences in item-nonresponse, and Couper et al. (2006) find higher item-nonresponse for the metric scale.

Scales can also provide a limited number of categorical options. I distinguish four main types of categorical scales: (1) *dichotomous scales* which only provide two substantive response options, typical dichotomous scales are yes–no and true–false; (2) *rating scales* which provide three or more categorical options; (3) *closed quantifiers* which are mainly used for objective variables such as the frequency of activities, omitting its response alternatives such scales become an open-ended quantifier; and (4) *branching scales* are used to simplify the respondents’ task when answering to long bipolar scales. Branching scales consist on dividing the response task in two steps. First, the respondents are asked about the direction of their judgment, i.e. neutral alternative versus the extreme sides of the bipolar scale. Second, they are asked about the extremity or intensity of their judgement on the selected side.

Rating scales require more interpretative efforts that may harm the consistency of the responses compared to dichotomous scales (Krosnick et al. 2005), whereas branching scales have been argued to be useful to explore the neutral alternatives and to provide large fully-labelled scales without a visual presentation (Schaeffer and Presser 2003). A handicap of closed quantifiers, compared to open quantifiers, is that the specified ranges inform respondents about the researcher’s knowledge of (or expectations about) the real world (Schwarz et al. 1985; Sudman and Bradburn 1983). In this direction, Revilla (2015, p. 236) for sensitive questions recommends providing “answer categories with high enough labels such that respondents do not feel that their behaviour is not normal”, and for non-sensitive questions “use labels following the expected population distributions such that respondents can use the middle of the scale as a reference point as to what is the norm, and evaluate their own behaviour as lower or higher than the average”. Looking at its impact on measurement quality, scales with 2-points usually perform worse than scales with more categories, with the exception of three-point scales (Krosnick 1991; Lundmark et al. 2016; Preston and Colman 2000). Only Alwin (2007) reports that dichotomous scales provide higher reliabilities than rating scales and absolute metric scales. On the contrary, some studies find evidence regarding branching scales producing higher measurement quality than rating scales (Krosnick 1991; Krosnick and Berent 1993). When rating scales are compared to continuous scales, like absolute metric scales or open-ended quantifiers, evidence is mixed: continuous scales are more reliable in Saris and Gallhofer (2007), but in Couper et al. (2001) and Miethe (1985) they provided higher item-nonresponse and lower reliability, respectively, than rating scales, and no differences between the two have been found on measurement quality by Koskey et al. (2013). Comparing rating to metric scales, the second appeared less reliable and leading to higher item-nonresponse in the studies of Cook et al. (2001), Couper et al. (2006) and Krosnick (1991), however, others find comparable impact between the two (Alwin 2007; Funke and Reips 2012; McKelvie 1978). Finally, Al Baghal (2014b) compares closed with open-ended quantifiers showing non-significant differences on measurement quality.

Overall, the decision on type of scale to provide has an impact on data quality and should be considered carefully when designing survey questions.

#### Scales’ length

The length of the scale is one of the key issues in scale development. As Krosnick and Presser (2010, p. 269) say, “the length of scales can impact the process by which people map their attitudes onto the response alternatives”.

The *minimum* and *maximum possible values* are used to evaluate the length of continuous scales. This characteristic has been fairly studied. Reips and Funke (2008) argue that differences on the length of metric scales may depend on the devices’ screen size and resolution, while, Saris and Gallhofer (2007) find a significant effect of the maximum possible value to answer in continuous scales on measurement quality.

The *number of categories* is used to evaluate the length of categorical scales. Among the characteristics of categorical scales, the number of categories is one of the most studied and complex design decisions: while a two-point scale allows only the assessment of the direction of the attitude, a three-point scale with a midpoint allows the assessment of both the direction and the neutrality, and even more categories allow the assessment of its intensity or extremity. Furthermore, while too few categories can fail to discriminate between respondents with different underlying opinions, too many categories may reduce the clarity of the meaning of the options and limit the capacity of respondents to make clear distinctions between them (Krosnick and Fabrigar 1997; Schaeffer and Presser 2003). The results regarding its impact on data quality are mixed. Most evidence suggest using more than 2-points to increase measurement quality (e.g. Andrews 1984). Some find evidence in favour of using 5–7-points (Komorita and Graham 1965; Rodgers et al. 1992; Scherpenzeel and Saris 1997). Others argue that options from 7 up to 10-points should be preferred (Alwin and Krosnick 1991; Lundmark et al. 2016; Preston and Colman 2000). Some others argue that even more categories, i.e. 11-points, can provide better measurements (Alwin 1997; Revilla and Ochoa 2015; Saris and Gallhofer 2007). Finally, others do not find differences across different number of points (Aiken 1983; Bendig 1954; Jacoby and Matell 1971; Matell and Jacoby 1971; McKelvie 1978). More recently, research has looked at the specific circumstances of the questions when evaluating the impact of the number of points. Some find, when distinguishing between item-specific and agree–disagree scales, that the quality does not improve for agree–disagree scales with more than 5-points (Revilla et al. 2014; Weijters et al. 2010) and for item-specific it goes up between 7 and 11-points (Alwin and Krosnick 1991; Revilla and Ochoa 2015). Similarly, Alwin (2007) argue that the optimal of points in a scale should be considered in relation to the scales’ polarity, and show that the use of 4-point scales improved the reliability in unipolar scales, while 2, 3 and 5-point scales improved the reliability in bipolar scales.

This summary has clearly shown that the length of the scale is a characteristic to consider.

### The scales’ labels

#### Verbal labels

Verbal labels are words used as a reference to clarify the meanings of the different scale points and its interval nature and reduce ambiguity (Alwin 2007; Krosnick and Presser 2010). Although it has been found that fully-labelling all points increases the cognitive effort of reading and processing all options (Krosnick and Fabrigar 1997; Kunz 2015). Studies about its effects on response style bias show that acquiescence is higher and extreme responding is lower with fully-labelled scales (Eutsler and Lang 2015; Moors et al. 2014; Weijters et al. 2010). Other studies about its impact show, higher reliability of end-points labelled scales compared to fully-labelled scales (Andrews 1984; Rodgers et al. 1992), while the majority show that labelling all points in the scale has a positive impact on reliability (Alwin 2007; Alwin and Krosnick 1991; Krosnick and Berent 1993; Menold et al. 2014; Saris and Gallhofer 2007). Thus, the impact on data quality is clear.

Usually a distinction between fully-labelled, partially-labelled and not at all labelled is made. However, there are multiple ways to design a scale partially-labelled and these should also be considered when assessing its effects on data quality. Thus, I propose the following distinction to cover the possible design choices in surveys: scales *not at all labelled, only labelled at the end*-*points, labelled at the end*- *and the midpoints, labelled at the end*- *and more points but not all,* and *fully*-*labelled.*

#### Verbal labels’ information

Verbal labels can provide different lengths and amounts of information. The more information is provided in the labels, the less information is needed in the request. Saris and Gallhofer (2007) distinguish between short labels or complete sentences and conclude that reliability improved when short labels instead of sentences are used. But still, more research is needed to assess the impact of this characteristic on data quality.

The length of a label does not actually provide sufficient advice on how to design them. For instance, even if using complete sentences may improve reliability are very long labels still preferable? It is for this reason, that I belief what affects data quality may be the amount of information provided in the label rather than its length. Thus, I propose the following differentiation. *Non*-*conceptual labels* require a previous specification of the type of measurement concept. For instance, the labels “Not at all” and “Completely” cannot be used without a previous specification of the concept like in the form of a question: “How satisfied are you with your job?”. Scales can otherwise provide *conceptual labels* like “Not at all satisfied”. Verbal labels can also provide information about the object and/or the subject under evaluation. An example of *objective label* would be “Not at all satisfied with my job”, and of *subjective label*, “I am not at all satisfied”. Finally, a *full*-*informative label* would be “I am not at all satisfied with my job”.

#### Quantifier labels

Two types of labels for closed quantifier scales can be distinguished. First, *vague quantifier labels* which are known to be prone to different interpretations, e.g. “often” can mean “once a week” for a respondent and “once a day” for another (Pohl 1981; Saris and Gallhofer 2014). In terms of its impact on data quality no clear conclusions can be extracted so far: Al Baghal (2014b) show that measurement quality is not affected with vague labels for closed quantifiers compared to open-ended responses, while Al Baghal (2014a) find higher levels of validity than in open-ended scales. Second, *closed*-*range* (or interval) *quantifier labels*, compared to vague quantifiers, are argued to be more precise and less prone to different interpretations (Saris and Gallhofer 2014). However, when providing closed-range quantifiers, respondents may use the frame of reference provided by the scale in estimating their own behaviour (Schwarz et al. 1985). Selecting unbiased ranges allowing respondents using the middle of the scale as a reference point is preferable (Revilla 2015). More research is needed to shed light towards whether the use of vague or closed-range quantifiers impacts or not data quality.

#### Fixed reference points

*Fixed reference points* are verbal labels used in a scale to prevent variations in the response functions and set no doubt about the position of the reference point on the subjective mind of the respondent (Saris 1988; Saris and Gallhofer 2014). For instance, the use of “always” and “never” can be fixed reference points on objective scales, and the words “not at all”, “completely”, “absolutely” and “extremely” for subjective scales. Usually, these are provided at the end-points of a scale. However, with closed-range quantifiers usually all labels are fixed reference points (e.g. “from 1 to 2 h”), and in bipolar scales, the midpoint alternative is also such. The use of fixed reference labels make the scale the same and comparable for all respondents (Saris and De Rooij 1988). Moreover, it has been proved to have a positive impact on improving measurements’ quality (Revilla and Ochoa 2015; Saris and Gallhofer 2007), and that when fixed reference points are not provided, respondents use different scales (Saris and De Rooij 1988).

#### Order of verbal labels

The ordering of verbal labels can be from *negative* (or passive)-*to*-*positive* (or active) or from *positive*-*to*-*negative*. The order of the verbal labels is an important characteristic since it provides an additional source of information to the respondents (Christian et al. 2007a). Moreover, scales ordered form positive-to-negative tend to provide more quick responses, which increases the chance that respondents do not processes all options consciously (Kunz 2015). Studies find that the order does impact measurement error and response style bias (Christian et al. 2007a, 2009; Krebs and Hoffmeyer-Zlotnik 2010; Saris and Gallhofer 2007; Scherpenzeel and Saris 1997).

#### Nonverbal labels

Nonverbal labels are numbers, letters or symbols instead of words attached to the options in the scale. The most commonly used are *numbers* and *symbols,* e.g. radio and checkbox buttons. Krosnick and Fabrigar (1997) suggest combining numerical and verbal labels. Similarly, others suggest that numbers may help respondents to decide whether the scale is supposed to be unipolar or bipolar (Schwarz et al. 1991; Tourangeau et al. 2007). However, respondents may take longer to submit an answer when numerical labels are provided since they are an additional source of information to process (Christian et al. 2009). Regarding its effect on data quality: Moors et al. (2014) show that scales without numbers and only verbal end-labels evoked more extreme responses than those with numbers, while Christian et al. (2009) and Tourangeau et al. (2000) conclude that response style is unaffected by the use or not of numbers in the scale. Thus, slightly more evidence points toward the fact that the choice of nonverbal labels does not affect data quality.

#### Order of numerical labels

Order of numerical labels can be from low-to-high or from high-to-low. From the few studies about its impact on response style that have been found, two of them conclude that, when negative numerical labels are provided compared to when all numbers are positive, the differences in the response distributions are significant (Schwarz et al. 1991; Tourangeau et al. 2007), while Reips (2002) concludes that it does not influence the answering behaviour of participants.

Since there is no classification, I propose the following distinction to account for the different choices in surveys: numerical labels ordered from *negative*-*to*-*positive,* from *positive*-*to*-*negative,* from *0*-*to*-*positive,* from *0*-*to*-*negative,* from *positive*-*to*-*0*, from *negative*-*to*-*0*, from *1 (or higher)*-*to*-*positive* or from *positive*-*to*-*1 (or higher).*

#### Correspondence between numerical and verbal labels

The order of numerical labels is of special relevance when these are combined with verbal labels. Correspondence between numerical and verbal labels refers to the extent to which the order of numerical labels matches with the order of verbal labels. Numerical labels should reinforce the meaning and the polarity of verbal labels (Krosnick 1999; Krosnick and Fabrigar 1997; O’Muircheartaigh et al. 1995; Schaeffer 1991; Schwarz et al. 1991). However, it should be considered that a more negative connotation is given to the label related to a negative number (Amoo and Friedman 2001; Schwarz and Hippler 1995). Following Saris and Gallhofer (2007) the level of correspondence is classified into: *high correspondence* which refers to combinations of numerical and verbal labels that match perfectly, e.g. a bipolar scale where numbers are ordered from -5 to +5 and verbal labels range from “Extremely bad” to “Extremely good” or a unipolar scale where numbers range from 0 to 10 and labels from “Not at all” to “Completely”; *low correspondence* which refers to combinations where the lower numbers are related to positive verbal labels or vice versa, e.g. a scale numbered from 0 to 10 and labelled from “Good” to “Bad”; and *medium correspondence* which refers to any other combination of numerical and verbal labels that matches the order of the labels: negative/low and positive/high but not perfectly. Among the little amount of empirical evidence found, only one study concludes that low correspondence do not impact the distribution of responses (Christian et al. 2007a), while two conclude that reliability improves with high correspondence between the verbal and the numerical labels in the scale (Rammstedt and Krebs 2007; Saris and Gallhofer 2007), i.e. there is an impact.

#### Scales’ symmetry

Symmetry is a specific characteristic of bipolar scales. *Symmetric scales* assure that the number of labels in bipolar scales is the same in the positive and in the negative side. *Asymmetric scales* assume previous knowledge about the population, otherwise it would be biased (Saris and Gallhofer 2014). However, its impact on measurement error is not clear: while Scherpenzeel and Saris (1997), for symmetric scales, find no effect (or very little) on reliability and validity, Saris and Gallhofer (2007) find a positive effect.

#### Neutral alternative

Neutral alternative is also a characteristic of bipolar scales, where the respondents are not forced to make a choice in a specific direction. Neutral alternatives can be provided implicitly or explicitly. *Explicit neutral alternatives* are usually labelled such as “neither A nor B”, while *implicit neutral alternatives* do not need to be labelled to understand its implicit neutral connotation, i.e. a bipolar scale with an uneven number of points, the midpoint will be considered neutral even if it is not labelled. Some argue that providing a neutral alternative can increase the risk of survey satisficing (Bishop 1987; Kulas and Stachowski 2009). Others argue that not providing a neutral point forces respondents to select an option which do not reflect the true attitudinal position (Saris and Gallhofer 2014; Sturgis et al. 2014). Finally, Tourangeau et al. (2004) argue that the neutral point in a scale can be interpreted as the most typical and use it to make relative judgements. Regarding the impact on response styles, studies find that including a neutral point increases acquiescence and lowers the propensity towards extreme responding (Schuman and Presser 1981; Weijters et al. 2010). In terms of its impact on measurements’ quality, most evidence suggest that providing the neutral impacts measurement quality (Alwin and Krosnick 1991; Malhotra et al. 2009; Saris and Gallhofer 2007; Scherpenzeel and Saris 1997). Only Andrews (1984) finds that the effect was very small.

#### “Don’t know” option

“Don’t know” (or “No opinion”) option is a non-substantive response alternative. These can also be implicit or explicit. An *implicit “don’t know” option* is an admissible answer not explicitly provided to the respondent, which requires an interviewer to record it. An *explicit “don’t know” option* can be directly provided as a different response alternative to the respondent. Providing an explicit “don’t know” option depends on whether researchers believe that respondents truly have no opinion on the issue in question (Dolnicar 2013; Kunz 2015). However, many authors argue that when the “don’t know” is provided this leads to incomplete, less valid and less informative data (Alwin and Krosnick 1991; Gilljam and Granberg 1993; Krosnick et al. 2002, 2005; Saris and Gallhofer 2014). Whether providing explicitly or implicitly a “don’t know” option impacts data quality is not clear: some authors show that providing it explicitly impacts data quality (Andrews 1984; De Leeuw et al. 2016; McClendon 1991; Rodgers et al. 1992), while others conclude that there is no support towards this impact (Alwin 2007; McClendon and Alwin 1993; Saris and Gallhofer 2007; Scherpenzeel and Saris 1997).

### The scales’ visual presentation

#### Types of visual response requirement

The type of visual presentation requires from the respondent higher or lower effort when responding. Following are the different types of visual response requirements distinguished in the literature: (1) *point*-*selection* is the most standard way to present scales, either a continuous line or categorical options are provided from which the respondent should point and select the desired choice; (2) *slider* is a type of linear implementation in which the respondent should move a marker to give a rating; (3) *text*-*box input* is a typing space where respondents can type in their answer; (4) *drop*-*down menu* shows the list of response options after clicking on the rectangular box, i.e. before clicking the respondent do not see the whole list of options and sometimes respondents have to scroll down to select the most desired option; and (5) *drag*-*and*-*drop* refer to the technique where respondents need to drag an element (e.g. the item or the response) to the desired position.

Comparing point-selection to sliders, the first are less demanding but also less fun and engaging (Funke et al. 2011; Roster et al. 2015). In this line, Cook et al. (2001) and Roster et al. (2015) compare sliders with radio buttons and find non-significant differences on reliability or item-nonresponse, respectively. The use of box format is closer to how questions are asked on the telephone, and do not provide a clear sense of the range of the options (Buskirk et al. 2015; Christian et al. 2009). Comparing the use of text-box input with the use of point-selection or sliders, some demonstrate that item-nonresponse and response style and are comparable across the three types (Christian et al. 2007b), while others show that there is an impact on item-nonresponse and response style between the three (Buskirk et al. 2015; Christian et al. 2009; Couper et al. 2006). Christian et al. (2007b) argue that drop-down menus are more cumbersome than text-box input when large number of options are listed. In this line, other authors argue that drop-down menus are more burdensome to respondents because they require an added effort to click and scroll (Couper et al. 2004; Dillman and Bowker 2001; De Leeuw et al. 2008; Reips 2002). Liu and Conrad (2016) compare drop-down menus with sliders or text-box input and find that item-nonresponse was non-significantly different. Similarly, when drop-down menus are compared to point-selection comparable results in terms of response style and item-nonresponse are found (Couper et al. 2004; Reips 2002). Finally, drag-and-drop provides higher item-nonresponse compared to point-selection and it is argued to prevent systematic response tendencies since respondent need more time to process what is the task they are required to do (Kunz 2015).

Overall, the evidence provided by these studies suggests that there is no impact on data quality depending on the type of visual response requirement.

#### Sliders’ marker position

Slider marker position is a specific characteristic of sliders. Markers can be placed at the *top*- *or left*-*side*, at the *bottom*- *or right*-*side*, at the *middle* or *outside* of a slider. A challenge when designing an slider is how to handle the starting position of the marker and identify non-respondents (Funke 2016). The impact of this characteristic on measurements’ error is not yet clear, since only one study looks at its effect on data quality and finds that higher nonresponse and higher response style bias occurred when the marker position was at the middle or the right-side of the slider compared to when the marker was placed at the left-side (Buskirk et al. 2015).

#### Scales’ illustrative format

Sometimes scales are presented using an illustrative format instead of using the traditional scales. Usual illustrative formats are *ladders* (or pyramids), to indicate levels of some aspect, and *thermometers*, to indicate degrees of feelings. Other illustrative formats can be *clocks* to indicate the timing of things, or *dials* to enter numerical values. The use of these types of scales usually require lengthy introductions and not all points can be labelled, but are useful to visually provide numerical scales with many points (Alwin 2007; Krosnick and Presser 2010; Sudman and Bradburn 1983). The few studies available suggest that this characteristic has an impact on data quality: thermometer scales provide less measurement quality than ladders or radio button scales (Andrews and Withey 1976; Krosnick 1991), ladder scales provide better measurement quality than traditional scales (Levin and Currie 2014) but lower validity compared to other illustrative formats (Andrews and Crandall 1975), and responses are significantly different whether a pyramid or an *onion* format are used (Schwarz et al. 1998).

#### Scales’ layout display

The scales’ layout display of the answer options can be *horizontal, vertical* or *nonlinear*. Nonlinear scales can provide, for instance, the answer options on different columns. Tourangeau et al. (2004, p. 372) argue that respondents usually expect, in vertically oriented scales, the positive points to appear first at the top. However, Toepoel et al. (2009, p. 522) argue that respondents read more naturally in a horizontal format. Two studies looked at the effect of scales’ layout display on response styles but they both find that whether presenting the scales in an horizontal, vertical or nonlinear layout provided significant differences on the responses (Christian et al. 2009; Toepoel et al. 2009), i.e. it has an impact.

#### Overlap between verbal and numerical labels

Overlap between labels is a characteristic considered by Saris and Gallhofer (2014) for which no relevance has been found while reviewing the literature. This characteristic intends to indicate whether the verbal labels used in a horizontal scale are *clearly connected* to one nonverbal label or they *overlap* with several of them. More research is needed on this characteristic to assess whether it is or not relevant to consider when designing visually presented scales.

#### Labels’ visual separation

Labels can be visually separated by adding more space between them, separating lines or the options in boxes. The aim of this is to provide a visual distinction between the labels in the scale. For instance, researchers may be interested in visually separating the “don’t know” option from the substantive responses to make a clear differentiation. However, Christian et al. (2009) and Tourangeau et al. (2004) argue that visually separating some of the labels may encourage respondents to select it more often. The impact on data quality is clear: De Leeuw et al. (2016) show that by separating the non-substantive option reduces item-nonresponse and provides higher reliability, Christian et al. (2009) and Tourangeau et al. (2004) show that separating the non-substantive option lead to significant differences on the responses while it do not happen when the midpoint is separated.

The current distinction in Saris and Gallhofer (2014) is whether the labels are separated within different boxes or not. However, given that I found more choices in the literature, I propose to distinguish between visually separating the *non*-*substantive option*, the *neutral option*, the *end*-*points, all points* or *none* of the points in the scale.

#### Labels’ illustrative images

Illustrative nonverbal labels can be used instead of or in combination with verbal and numerical labels when they are provided visually to the respondent. Usual illustrative labels are: *feeling faces* (also called smileys) which attach images of different face expressions (e.g. from sad to happy). They are easy to format and they attract the attention of the respondents (Emde and Fuchs 2013). Moreover, they have the advantage of being easier to identify by respondents than verbal labels because they eliminate the barrier of mapping feelings into words (Kunin 1998). Its effect on data quality indicate that there is no impact: while Derham (2011) shows that nonresponse is significantly higher in faces scales compared to sliders and point-selection scales, Andrews and Crandall (1975), Emde and Fuchs (2013) show that the differences in the responses between smiley scales and radio button are non-significant.

For the sake of completeness and to capture the different formats found in the literature I propose to distinguish two other types labels’ illustrative images: *other human symbols*, like thumbs and manikins, and *other nonhuman symbols,* like stars or harts.

## Conclusions

This paper provides a complete and updated classification of the characteristics and its possible design choices considered in the literature when designing forced-choice, closed and ordinal response scales. This classification has been summarized in Table 1 together with the main conclusion of the literature review, which indicate whether evidence has been shown in the literature of each characteristics’ impact on data quality.

Three main limitations of this study should be kept in mind: First, to assess whether there is an impact or not on data quality, I did not consider the different sample sizes or the power of the studies. I considered the absolute amount of studies. Further research, could provide weights to the different studies. Second, it is likely that publication bias in favour of studies which found an effect of a certain characteristic is present, i.e. the number of characteristics which have an impact may be overestimated. Third, I did not aim to provide information to improve the design of response scales. Thus, the results on the impact are provided independently of its positive or negative effect.

From Table 1 the following main conclusions can be extracted:

1. 11 characteristics have an impact on data quality: the scales’ evaluative dimension, the type of scale, the length of the scales, the use of verbal labels, the use of fixed reference points, the order of numerical labels, the correspondence between numerical and verbal labels, the use of a neutral alternative, the scales’ illustrative format, the visual layout display of the scales, and the labels’ visual separation.
2. 4 characteristics do not have an impact on data quality: the order of the verbal labels, the use of nonverbal labels, the type of visual response requirement, and the labels’ illustrative images.
3. Further research is needed for 8 characteristics: to know whether the scales’ polarity, the agreement between concept and the scale’s polarity, the information provided by verbal labels, the quantifier labels, the scales’ symmetry, the use of a “don’t know” option, the slider marker position, and the overlap between verbal and numerical labels have or not an impact on data quality.

What is clear from the large body of research presented here and its often mixed results is that characteristics interact with each other, e.g. usually scales with more points are partially labelled. Thus, researchers should account for the effects driven by the overall design of the survey question, when assessing how to optimally decide upon a characteristic. That is in line to what Cox III (1980, p. 418) already concluded for the optimal number of categories: “there is no single number of response alternatives for a scale which is appropriate under all circumstances”.

The results presented in this paper provide on the one hand a source for researchers that want a complete list of characteristics and its possible design choices for closed and ordinal scales, and on the other hand, a detailed summary of the literature that refer to the impact of each characteristic on data quality.

Finally, further research should provide the same summary for other characteristics related to the design of survey questions, such as the design of the request for an answer or the overall visual presentation of the survey question.

## Appendix

See Tables 2 and 3.

**Table 2:** Saris and Gallhofer’s list of response scale characteristics and choices

Characteristics	Design choices
Response scale: basic choice	More than 2 categories scale
	Two-category scale
	Numerical open-ended scale
	Magnitude estimation
	Line production
	More steps procedures
Number of categories (categorical)	[Enter value]
Maximum possible value (continuous)	[Enter value]
Labels of categories	No labels
	Partially-labelled
	Fully-labelled
Labels with short text or complete sentences	Short text
	Complete sentences
Order verbal labels	First label negative
	First label positive
Correspondence between numerical and verbal labels	High correspondence
	Medium correspondence
	Low correspondence
Range of the used scale	Bipolar
	Unipolar
Range correspondence	Both bipolar
	Both unipolar
	Concept bipolar/Scale unipolar
Symmetry of response scale	Symmetric
	Asymmetric
Neutral category	Present
	Absent
Number of fixed reference points	[Enter value]
“Don’t know” option	Present
	Only registered
	Absent
Horizontal or vertical scale	Horizontal
	Vertical
Overlap between verbal and numerical labels	Present
	Text clearly connected to categories
Numbers or letters before answer categories	Numbers
	Letters
	Neither
Scale with only numbers or numbers in boxes	In boxes
	Not in boxes

**Table 3:** Literature review summary of findings by theoretical and empirical argumentations

Characteristics	Design choices	Theoretical arguments	Empirical evidence on data quality
*Characteristics of the response scales’ conceptualization*			
Scales’ evaluative dimension	Agree–disagree (AD) Item-specific (IS)	(Brown 2004): AD scales are clearer to interpret than vague or closed-range quantifier scales (Krosnick 1999): people simply choose to agree because it seems like the commanded and polite action to take (Krosnick et al. 2005): to eliminate acquiescence avoid AD scales (Kunz 2015): AD scales are more difficult to understand and map the appropriate judgement (Saris et al. 2010): AD more acquiescence because of its usual presentation in batteries (Schaeffer and Presser 2003): AD simpler to conduct	(Alwin 2007): the reliability of AD scales is lower compared to IS scales [Wiley–Wiley reliability] → YES (Billiet and McClendon 2000): Acquiescence is found in AD scales [Acquiescence bias through SEM factor] → YES (Krosnick 1991): AD scales lead to lower reliabilities than IS [Pearson product-moment test-rest correlations] → YES (Revilla and Ochoa 2015): AD scales have much lower quality than IS [True-score MTMM reliability and validity] → YES (Saris and Gallhofer 2014): AD scales have lower quality than IS [True-score MTMM reliability and validity] → YES (Saris et al. 2010): IS scales have higher quality than AD [True-score MTMM reliability and validity] → YES
Scales’ polarity	Bipolar Unipolar	(Kunz 2015): a disadvantage of bipolar scales is that respondents are reluctant to choose negative responses	(Alwin 2007): unipolar scales have somewhat higher reliabilities than bipolar scales [Wiley–Wiley reliability] → YES
Concept-Scale polarity agreement	Both bipolar Both unipolar Bipolar concept with Unipolar scale Unipolar concept with Bipolar scale	(Rossiter 2011): not distinguish between unipolar and bipolar leads to stupid misinterpretations; unipolar attributes should not be measured with bipolar scales	(Saris and Gallhofer 2007): the impact of using unipolar scales for bipolar concepts is not significantly lowering reliability and increasing validity [True-score MTMM reliability and validity] → NO (van Doorn et al. 1982): differences in the response distributions are clear [Response style through distribution comparison] → YES
*Characteristics of the type of response scale and its length*			
Type of response scales	Absolute open-ended quantifier Relative open-ended quantifier Relative metric Absolute metric Dichotomous Rating Closed quantifiers Branching	(Hjermstad et al. 2011): metric scales are comparable to categorical scales; the type of scale is not the most important but the conditions related to them (Krosnick et al. 2005): dichotomous scales are clearer in meaning and require less interpretative efforts which can harm consistency compared to rating scales (Krosnick and Fabrigar 1997): relative open-ended scales (or magnitude scaling) are a difficult method to administer which only reveals ratios among stimuli and not absolute judgments (Liu and Conrad 2016): respondents are more likely to provide rounded answers in 101 metric scales, as an easy way out (Revilla 2015): the closed-range quantifier labels provided can influence their results if they do not represent the population distribution (Saris and Gallhofer 2014): line production (or relative metric) scales are better than relative open-ended quantifiers because rounding is avoided (Schaeffer and Bradburn 1989): magnitude estimates (or relative open-ended quantifiers) have problems related to the appropriate standard and recoding into categorical distinctions (Schaeffer and Presser 2003): branching has the advantage to provide large number of categories not visually (Schwarz et al. 1985): closed-range informs the respondent about the researcher expectations and adds systematic bias in respondent’s reports and related judgements compared to absolute open-ended formats (Sudman and Bradburn 1983): better use open quantifiers than closed quantifiers for numerical answers to avoid misleading the respondent (Tourangeau et al. 2000): round answers in open-ended quantifiers may be a signal of the unwillingness to come up with a more exact answer and introduce systematic bias, in continuous scales	(Al Baghal 2014a): numerical open ended are as accurate as vague-closed options [Rank-order correlations and regression slopes] → NO (Alwin 2007): rating scales have higher reliabilities than dichotomous but comparable to metric scales [Wiley–Wiley reliability] → YES (Cook et al. 2001): metric scale less reliable than radio button [Score reliability] → YES (Couper et al. 2006): metric scales suffer more missing data than categorical or open-ended quantifier [Item-nonresponse] → YES (Funke and Reips 2012): metric scales are comparable to 5p scales on item-nonresponse [Item-nonresponse] → NO (Koskey et al. 2013): absolute open-ended scales are comparable to rating scales on reliability [Cramer’s V reliability] → NO (Krosnick 1991): metric scales have lower reliability than rating scales; lower reliabilities when using dichotomous scales; branching provides higher reliabilities than rating scales [Pearson product-moment test–retest correlations] → YES (Krosnick and Berent 1993): branching improves reliability compared to no branching (rating scale) [Item reliability] → YES (Liu and Conrad 2016): non-significant differences on item-nonresponse between absolute open ended, rating scale or metric [Item-nonresponse] → NO (Lundmark et al. 2016): dichotomous less valid than rating scales [Concurrent validity] → YES (McKelvie 1978): no difference on reliability or validity between metric and rating scale [Test retest reliability and Test validity] → NO (Miethe 1985): magnitude scaling less credible in terms of reliability compared to rating scales [Test–retest reliability] → YES (Preston and Colman 2000): 2p scales less reliable and valid [Test retest reliability, Cronbach alpha and Criterion validity] → YES (Saris and Gallhofer 2007): open-ended quantifiers and metric scales have significantly higher reliability but lower validity than rating scales [True-score MTMM reliability and validity] → YES
Response scales’ length	Minimum possible value Maximum possible value Number of categories	(Alwin 2007): the optimal number of points in a scale should be taken into consideration in relation to the polarity of the scale (Cox III 1980): there is no single number of response alternatives for a scale which is appropriate under all circumstances (Krosnick and Fabrigar 1997): optimal is a complex decision to few categories may compromise the information gathered, too long compromises the clarity of meaning (Reips and Funke 2008): optimal length of continuous scales depends on the size of the device screen (Schaeffer and Presser 2003): more categories compromise discrimination and limit the capacity of respondents to make finer distinctions between the options	(Aiken 1983): reliabilities remained constant despite changing the number of categories [Internal consistency reliability] → NO (Alwin 1997): 11p scales more reliable than 7p [True Score MTMM reliability] → YES (Alwin 2007): the use of 4p scales improves reliability in unipolar scales, while the reliability in bipolar scales is higher for 2, 3 and 5p and lowest for 7p. [Wiley–Wiley reliability] → YES (Alwin and Krosnick 1991): no differences between AD with 2 and 5p, IS reliability increases from 3 to 9p, but no differences between 7 to 9p [Proportion of variance attributed to true attitudes] → YES (Andrews 1984). The biggest effect on data quality. More categories better. 3p is worse than 2p [MTMM validity, method effect and residual error] → YES (Bendig 1954): reliability independent of the number of scale categories [Test reliability] → NO (Jacoby and Matell 1971): reliability and validity are independent of the number of points [Test retest reliability, concurrent validity and predictive validity] → NO (Komorita and Graham 1965): reliability increases with the number of points up to 6p [Cronbach alpha] → YES (Lundmark et al. 2016): validity higher in 7p and 11p points than 2p [Concurrent validity] → YES (Matell and Jacoby 1971): reliability independent of the number of points [Internal consistency and Test retest reliability] → NO (McKelvie 1978): validity is slightly better on 7p rather than 11p, reliability unaffected scale [Test retest reliability and Test validity] → NO (Preston and Colman 2000): reliability lower for 2, 3, 4p, higher for 7, 8, 9, 10p, decreases with more than 10p [Test–retest reliability] → YES (Revilla and Ochoa 2015): 11p affects positively the quality of IS scales [True-score MTMM reliability and validity] → YES (Revilla et al. 2014): quality does not improve with more than 5p for AD scales [True-score MTMM reliability and validity] → YES (Rodgers et al. 1992): the number of points has the biggest effect on validity; use at least 5 to 7p, better quality [MTMM construct validity] → YES (Saris and Gallhofer 2007): reliability can be improved by using more categories (11p) without decreasing validity; [True-score MTMM reliability and validity] → YES (Saris and Gallhofer 2007): the maximum value of a continuous scale has a significant effect on reliability or validity [True-score MTMM reliability and validity] → YES (Scherpenzeel and Saris 1997): highest validity with 4, 5 or 7p [True-score MTMM validity] → YES (Weijters et al. 2010): 5 AD points reduces extreme response style [Extreme Response Style through log odds] → YES
*Characteristics of the response scales’ labels*			
Verbal labels	Fully-labelled End-points and more points labelled Endand midpoints labelled End-points only labelled Not labelled	(Alwin 2007): labels reduce ambiguity in translating subjective responses to scales’ options (Krosnick and Fabrigar 1997): verbal labels suffer from language ambiguity and are more complex to hold in memory, label only the endpoints are less cognitively demanding than fully labelling; verbal labels are more natural form of expression than numbers and labelling all points can help to clarify the meaning of numbers (Krosnick and Presser 2010): verbal labels are advantageous because they clarify the meanings of the scale points while reducing the respondent burden (Kunz 2015): labelling may increase the cognitive effort required to read and process all options, while clarifying the meaning of them	(Alwin 2007): fully labelled increases reliability significantly compared to only labelling the endpoints. [Wiley–Wiley reliability] → YES (Alwin and Krosnick 1991): fully labelled increases reliability [Proportion of variance attributed to true attitudes] → YES (Andrews 1984): data quality is below average with all categories labelled [MTMM validity, method effect and residual error] → YES (Eutsler and Lang 2015): Fully labelled produces less extreme responses [Extreme response bias through distribution comparison] → YES (Krosnick and Berent 1993): full verbal labelling improve reliability [Item reliability] → YES (Menold et al. 2014): Fully labelled scales have higher reliabilities than when only the endpoints are labelled [Guttman’s lambda] → YES (Moors et al. 2014): end labelling evokes more extreme responses [Extreme response bias through latent class factor] → YES (Rodgers et al. 1992): non-verbal alternatives have lower random error [MTMM construct validity] → YES (Saris and Gallhofer 2007): The use of labels increase reliability significantly [True-score MTMM reliability and validity] → YES (Weijters et al. 2010): higher acquiescence and lower extreme scores when all categories are labelled [Acquiescence and Extreme response bias through log odds] → YES
Verbal labels’ information	Non-conceptual Conceptual Objective Subjective Full-informative	–	(Saris and Gallhofer 2007): reliability reduced by having large labels [True Score MTMM reliability] → YES
Quantifier labels	Vague Closed-range	(Brown 2004): AD scales are clearer to interpret than vague quantifiers (Pohl 1981): it is not clear what exactly word set provides better equal interval scaling (Revilla 2015): closed-range should provide enough labels such that respondents do not feel that their behaviours are not normal (Saris and Gallhofer 2014): vague are prone to different interpretations than closed (Schwarz et al. 1985): respondents use the labels like “usual” as standards of comparison and seem reluctant to report behaviours that are unusual in the context of the scale	(Al Baghal 2014b): vague quantifiers display higher levels of validity than numeric open-ended quantifiers [Predictive validity] → YES (Al Baghal 2014a): vague are equal or better than open-ended quantifiers [Rank-order correlations and regression slopes] → NO
Fixed reference points	Number of fixed reference points	(Saris and De Rooij 1988): the reference points should add no doubt of its position on the subjective scale of the respondents (Saris and Gallhofer 2014): reference points are necessary to assure that respondents are using the same underlying scale	(Revilla and Ochoa 2015): the use of two fixed reference points increases slightly measurement quality [True-score MTMM reliability and validity] → YES (Saris and De Rooij 1988): differences are due to the freedom respondents have when no fixed reference points are stablished [Response bias through distribution comparison] → YES (Saris and Gallhofer 2007): fixed reference points have a positive and significant effect on reliability and validity [True-score MTMM reliability and validity] → YES
Order verbal labels	From negative-to-positive (N-P) From positive-to-negative (P-N)	(Christian et al. 2007b): responses vary depending on the order since it provides an addition source of information (Kunz 2015): P-N scales may tempt respondents to rush through a set of items at a faster pace	(Christian et al. 2007b): the order of the verbal labels does not provide significant differences on responses [Response style through distribution comparison] → YES (Christian et al. 2009): no primacy effect found by varying the order of the verbal labels [Satisficing bias through distribution comparison] → YES (Krebs and Hoffmeyer-Zlotnik 2010): more positive answers (primary effect) on P-N, non-significant evidence in the N-P format [Satisficing bias through distribution comparison] → YES (Saris and Gallhofer 2007): the order does not have a significant impact on measurement quality [True-score MTMM reliability and validity] → NO (Scherpenzeel and Saris 1997): order had little or no effect on validity and reliability [True-score MTMM reliability and validity] → NO
Nonverbal labels	Numbers Letters Symbols None	(Christian et al. 2009): adding numbers provides an additional source of information to process by the respondents before submitting an answer (Krosnick and Fabrigar 1997): numeric labels more precise and easier but have no inherent meaning (Tourangeau et al. 2007): numbers help respondents to decide whether the scale is supposed to be unipolar or bipolar (Schwarz et al. 1991): use numeric labels to disambiguate the meaning of scale verbal labels. 0 to10 numbers suggest the absence or presence of an attribute, while -5 to 5 suggest that the absence corresponds to 0 whereas the negative values refer to the presence of its opposite	(Christian et al. 2009): response style is unaffected when using scales with or without numbers [Satisficing bias through distribution comparison] → NO (Moors et al. 2014): scales with no numbers evoke more extreme responding than with numbers [Extreme response bias through latent class factor] → YES (Tourangeau et al. 2000): scales with no numbers are comparable to those with positive numbers [Response style through distribution comparison] → NO
Order numerical labels	Negative-to-positive Positive-to-negative 0-to-positive 0-to-negative Positive-to-0 Negative-to-0 1 (or higher)-to-positive Positive-to-1 (or higher)	–	(Schwarz et al. 1991): differences are significant when a scale is presented with 0 to10 values or with -5 to 5 [Response style through distribution comparison] → YES (Tourangeau et al. 2007): differences are significant when negative numerical labels are provided in comparison to when all are positive [Response style though distribution comparison] → YES (Reips 2002): different numerical labelling do not seem to influence the answering behaviours of participants [Response style through distribution comparison] → NO
Correspondence between numerical and verbal labels	High Medium Low	(Amoo and Friedman 2001): more negative connotation is attached to negative numbers than positive with the same verbal label (Krosnick 1999): use only verbal labels or use numbers that reinforce the meanings of the words (Krosnick and Fabrigar 1997): numbers should be selected carefully to reinforce the meaning of the scale points (O’Muircheartaigh et al. 1995): numeric and verbal labels should provide bipolar/unipolar framework to the respondent (Schaeffer and Presser 2003): when bipolar verbal labels are combined with bipolar numeric labels they would reinforce each other to appear clearer to respondents, however bipolar numeric labels move responses toward the positive end (Schwarz and Hippler 1995): a verbal scale with a negative numeric value suggest a more negative interpretation of the verbal scale anchor and results in more positive responses along the scale (Schwarz et al. 1991): match numeric values with the intended conceptualization of the unior bipolar dimension, numbers should not be selected arbitrarily because respondents use them to communicate intended meanings	(Christian et al. 2007b): low correspondence does not impact substantially the responses [Response style through distribution comparison] → NO (Rammstedt and Krebs 2007): lower reliabilities when the lower numbers correspond to higher positive labels [Test–retest reliability] → YES (Saris and Gallhofer 2007): low correspondence lowers significantly reliability [True-score MTMM reliability] → YES
Scales’ symmetry	Symmetric Asymmetric	(Saris and Gallhofer 2014): an asymmetric scale presupposes knowledge about the opinion of the sample, otherwise is biased	(Saris and Gallhofer 2007): symmetric scales have a positive effect on reliability and validity [True-score MTMM reliability and validity] → YES (Scherpenzeel and Saris 1997): reliability and validity are slightly higher for asymmetric scales [True-score MTMM reliability and validity] → NO
Neutral alternative	Explicit Implicit Not provided	(Bishop 1987): midpoints attract respondents under uncertainty (Kulas and Stachowski 2009): midpoints are used when respondents are undecided, misunderstanding the item, when their response is conditional or when they have a neutral opinion (Saris and Gallhofer 2014): used to not force people to make a choice on a specific direction (Sturgis et al. 2014): people do appear to have positions which are neutral; omitting will force these individuals to select an option which does not reflect the true opinion (Tourangeau et al. 2004): respondents can interpret de midpoint in a scale as the most typical and use it as reference point	(Alwin and Krosnick 1991): Midpoints lower reliability, more valuable in 7 point scales [Proportion of variance attributed to true attitudes] → YES (Andrews 1984): midpoint had only slight effect on data quality [MTMM validity, method effect and residual error] → NO (Malhotra et al. 2009): midpoint reduces validity [Criterion validity] → YES (Saris and Gallhofer 2007): not providing a neutral category improves significantly both reliability and validity [True-score MTMM reliability and validity] → YES (Scherpenzeel and Saris 1997): explicit midpoint has no effect on reliability but a higher validity [True Score MTMM reliability and validity] → YES (Schuman and Presser 1981): offering the middle alternative increases the proportion of respondents in that category [Response style through distribution comparison] → YES (Weijters et al. 2010): midpoint increases acquiescence and lowers extreme responses [Acquiescence and Extreme response bias] → YES
“Don’t know” (DK) option	Explicit Implicit Not provided	(Alwin and Krosnick 1991): DK may be selected because of truly not having an attitude, lack of motivation, wish to avoid giving an answer or are uncertain of which exact point represents best their opinion (Dolnicar 2013): if some respondents cannot answer the question, offer explicit DK (Gilljam and Granberg 1993): explicit DK increases the likelihood of false negatives (Krosnick et al. 2002): providing DK leads to less valid and informative data than omitting it (Krosnick et al. 2005) DK provision encourages respondents to not provide undesirable or unflattering opinions (Kunz 2015): DK option should be explicitly provided if there is a good reason to believe that respondents truly have no opinion on the issue in question (Saris and Gallhofer 2014): explicit DK leads to incomplete data, better use implicit DK	(Alwin 2007): Providing an explicit DK option has a comparable reliability to not providing it [Wiley–Wiley reliability] → NO (Andrews 1984): explicit DK leads to higher data quality [MTMM validity, method effect and residual error] → YES (De Leeuw et al. 2016): Explicit DK increases missing data and lowers reliability. Implicit DK lowers missing data and increases reliability [Item non-response and Coefficient alpha] → YES (McClendon 1991): explicit DK does not reduce acquiescence or recency responses [Acquiescence and Satisficing bias] → YES (McClendon and Alwin 1993): no support towards offering DK to improve reliability [True-score reliability] → NO (Rodgers et al. 1992): lower validities when offering DK explicitly [MTMM construct validity] → YES (Saris and Gallhofer 2007): The provision of the DK option does not have a significant effect on measurement quality [True-score MTMM reliability and validity] → NO (Scherpenzeel and Saris 1997) DK explicit or implicit does not affect reliability or validity [True-score MTMM reliability and validity] → NO
*Characteristics of the response scales’ visual presentation*			
Types of visual response requirement	Point-selection Slider Text-box input Drop-down menu Drag-and-drop	(Buskirk et al. 2015): box format does no give a clear sense of the range of the options (Christian et al. 2007a): numeric text-box input better because drop-down menus are more cumbersome when large number of possible options are listed (Christian et al. 2009): box format is closer to how questions are asked on telephone, where the visual display is not provided (Couper et al. 2004): drop boxes require added effort from respondents who have to click and scroll simply to see the answer options (De Leeuw et al. 2008): drop-down menus are more burdensome for respondents (Dillman and Bowker 2001): respondents are more frustrated with drop-down menus as it requires a two-step process (Funke et al. 2011): more demanding requires more hand–eye coordination than point-selection and provides problems to identify non-substantive responses (Kunz 2015): drag and drop may prevent systematic response tendencies since respondents need to spend more time (Reips 2002): hand movement is longer than for other types of scales (Roster et al. 2015): sliders are more fun and engaging and produce better data than point-selection scales	(Buskirk et al. 2015): differences on selecting the lowest, middle or highest options and in missing data between sliders, radio button scales and box format [Satisficing bias and Item-nonresponse] → YES (Christian et al. 2007b): responses are comparable between point-selection and number box scales [Response style through distribution comparison] → NO (Christian et al. 2009): Box entry has a significant impact on responses compared to point-selection [Response style bias through distribution comparison] → YES (Cook et al. 2001): sliders show no difference compared rating scales on reliability [Score reliability] → NO (Couper et al. 2004): nonresponse was comparable between drop-down menu and point-selection [Item-nonresponse] → NO (Couper et al. 2006): more missing data in the slider than in the radio button or text input scale [Item-nonresponse] → YES (Kunz 2015): drag-and-drop scales suffered from higher item-nonresponse compared to radio button scales [Item-nonresponse] → YES (Liu and Conrad 2016): item-nonresponse is nonsignificantly different compared to drop-down and text-box input [Item-nonresponse] → NO (Reips 2002): drop-down menus do not influence on the answering behaviours compared to radio button scales [Response style through distribution comparison] → NO (Roster et al. 2015): response rates between sliders and radio-button scales are non-significantly different [Item-nonresponse] → NO
Sliders’ marker position	Left/Bottom Right/Top Middle Outside	(Funke 2016): a drawback of sliders is item-nonresponse is difficult to identify	(Buskirk et al. 2015): more nonresponse, middle and higher response options selection for middle and right marker position compared to left marker [Satisficing bias and item-nonresponse] → YES
Scales’ illustrative format	Ladder Thermometer Other None	(Alwin 2007): offering a thermometer scale usually requires lengthy introductions (Krosnick and Presser 2010): thermometers and ladders may not be good measuring devices because all points cannot be labelled (Sudman and Bradburn 1983): use thermometers, ladders, telephone dials and clocks for numerical scales with many points	(Andrews and Crandall 1975): ladder scales obtained lower validity than other types of scales [Construct validity] → YES (Krosnick 1991): reliability is higher for a rating scale than for the feeling thermometer [Pearson product-moment test–retest correlations] → YES (Levin and Currie 2014): the ladder scale provided better reliability and validity scores than other scales [Pearson correlations and convergent validity] → YES (Schwarz et al. 1998): responses are significantly different whether a pyramid or an onion format is used [Response style through distribution comparison] → YES
Scales’ layout display	Horizontal Vertical Nonlinear	(Toepoel et al. 2009): respondents are more willing to read option in the horizontal format because they first read horizontally and then vertically (Tourangeau et al. 2004): vertical scales imply more positive options at the top	(Christian et al. 2009): responses to nonlinear layout compared to vertical were significantly different [Response style through distribution comparison] → YES (Toepoel et al. 2009): presenting the options in a horizontal or vertical layout results in different response distributions [Response style through distribution comparison] → YES
Overlap between verbal and numerical labels	Overlap present Text clearly connected to categories	NS	NS
Labels’ visual separation	Non-substantive options Neutral options End-points All options None	(Christian et al. 2009): visual separation of labels may encourage respondents to select it and may take longer for respondents to process than when all labels are evenly spaced (Tourangeau et al. 2004): separation calls the attention of the separated option	(De Leeuw et al. 2016): clearly separating the DK option from the substantive responses reduces missing data and produced higher reliability [Item nonresponse and Coefficient alpha] → YES (Christian et al. 2009): separation of the non-substantive option leads to significant different responses, separation of the midpoint does not lead to significant differences [Response style through distribution comparison] → YES (Tourangeau et al. 2004): separation of non-substantive options affected the distribution of answers [Response style through distribution comparison] → YES
Labels’ illustrative images	Feeling faces Other human symbols Non-human symbols None	(Emde and Fuchs 2013): faces scales are easy to format and attract the attention and increase respondents’ enjoyment (Kunin 1998): Faces scales have the advantage of eliminating the necessity for translating feelings into words, faces are easier to identify by respondents than words	(Andrews and Crandall 1975): comparable validity between faces scales and rating scales [Construct validity] → NO (Derham 2011): the emoticon scale presented significantly higher no answers than slider or point-selection scales [Item-nonresponse] → YES (Emde and Fuchs 2013): non-significant differences in the responses between the smiley scales and the radio button design [Response style through distribution comparison] → NO
